# The Image of Pharma

**DOI:** 10.4103/0973-1229.32148

**Published:** 2007

In this section, we shall look into issues like code of conduct for industry, voluntary moratorium on spending, why pharma image stinks, the recent goof up about Trasylol (Aprotinin) and what AMCs and medical and research institutes can do to salvage their image vis-à-vis industry collaboration.

## Voluntary Moratorium And Code Of Conduct

It is heartening to note that a recent news item reports on a voluntary moratorium by a major chunk of pharma players in India. They are members of the Organisation of Pharmaceutical Producers of India (OPPI) representing companies that control nearly two-thirds of the medicine market. This is a recent (Jan 17, 2007) headline in a prominent Indian daily *(TOI*):

Drug companies ban freebies to doctors (Mukherjee, 2007).

This news reports a decision to curb expenses on foreign jaunts of doctors, gifts in cash or kind, shopping, entertainment and many other major expenses in line with, what it calls, ‘international standards that support self-regulation through compliance’ (ibid).

In this connection, it may be pertinent to note that even companies like Pfizer are reported to be planning to cut their work force by 20% in the US, mainly because it has become like a dinosaur. Moreover, doctors have little time to listen to them, little latitude to prescribe and equally importantly, *‘many hospitals and medical schools increasingly frown on doctors enjoying free dinners and trips from pharmaceutical firms, which once were commonplace in the business. A steady stream of state and federal investigations into drug-marketing practices also has had a chilling effect’* (Kennedy, 2006). In other words, there is scope for cost cutting if only administrators in colleges, hospitals and the state get into the act. Glaxo is likely to follow suit (Cohen, 2006), since *‘company after company has admitted that its ROI on multiplexed sales calls (multiple calls to the same practice) has eroded and company after company has quietly reacted to this erosion with measured, targeted reductions in the number of calls per physician’* (Ibid). In other words, while physicians can heave a sigh of relief from the constant deluge of medical reps, patients and other payers can expect some trickle effect in the form of cost-cutting of escalating drug prices.

Coming to the present Indian attempt, it is part of an initiative to draw up a code of conduct that companies have agreed to apply to themselves. Called the OPPI Code of Pharmaceutical Marketing Practices January 2007, it is drawn up by the OPPI (OPPI Home, 2007), which claims to be not only an industry association but also a scientific and professional body. And to show that they mean business, the Code comes into immediate effect, from January 2007 itself.

While it is presumptuous to claim that what we wrote had any impact on what pharma bosses decide, may we mention that the Academia-Industry Symposium of the *MSM* (Singh and Singh, 2005; 2005-2006) did reach the top pharma bosses in India. And while there is no way of knowing whether they served as an eye-opener or not, it is heartening to note that some steps in the direction we espoused have been taken, albeit belatedly. For this, the industry deserves accolades.

## Pharma Image Stinks: Why?

Attempts at self-regulation and damage control of credibility are also important in the light of realizations dawning on pharma bosses that their image stinks. Savour a relatively recent (22nd Oct 2006) headline:

Pfizer boss admits: our image stinks (Dixon, 2006)

This is a report based on Jack Watters′ statement that ‘*the drug industry suffers “crippling cynicism’ from the public about its motives and the huge profits it makes’ and* pharma companies were partly to blame since they had *‘failed to promote the positive contributions they make to society’* (Dixon 2006). Watters is Pfizer's Vice-President of medical and regulatory affairs for Europe, Latin America, Africa and the Middle East.

Now, why does this image stink? It's not only because it has failed to promote its positive contributions to society. It's because of its own sins of omission and commission. What makes us say this? Savour another headline just a little more than a fortnight before the one quoted above (3^rd^ Oct 2006):

**Bayer admits withholding drug study from FDA (Harrison, 2006)**

In this, Bayer has admitted it *mistakenly* failed to disclose data to the FDA from a retrospective study that analyzed the risks of its bleeding prevention drug Trasylol in patients undergoing coronary artery bypass surgery. The explanation by the company: *‘this data was not shared immediately because it was preliminary in nature and raised significant questions on the study population, outcomes and methodology’* (ibid). Now, that this explanation does not wash is obvious from an earlier headline that appeared eight months ago, on 3^rd^ February 2006, in which the problem with the drug was already clearly reported:

**Bayer: cardiovascular concerns could run deep (Pharmaceutical Business Review Online: PBR Online, 2006)**

This *PBR* Online article mentions Bayer could face legal action over safety concerns related to Trasylol, following the 2006 release of the content of a 4,374-patient assessment by the Ischemia Research and Education Foundation (IREF Home, 2007), whose researchers reported in the *NEJM* that Trasylol (aprotinin) more than doubled the risk of patients suffering renal failure requiring dialysis, was associated with a 55% increase in the risk of myocardial infarction or heart failure and a 181% increase in the risk of stroke or encephalopathy (Mangano *et al*, 2006). In contrast, both the generically available drugs used for the same condition hitherto, namely aminocaproic acid and tranexamic acid, were not associated with increased risk of renal, cardiac or cerebral events.

The paper concludes that the association between aprotinin and serious end-organ damage indicates that continued use was not prudent. In contrast, the less expensive generic medications aminocaproic acid and tranexamic acid were safe alternatives.

In an extension to this paper, the same authors report recently in the *JAMA* (6th Feb 2007) the results of an Observational study of mortality conducted between November 11, 1996 and December 7, 2006, following index hospitalization in 69 medical centers. Here, survival was prospectively assessed at 6 weeks, 6 months and annually for 5 years after CABG surgery among 3876 patients enrolled in a 62-center international cohort study (Mangano *et al,* 2007). They found Aprotinin was associated with significant increase in mortality compared to control, whereas neither aminocaproic acid, nor tranexamic acid was so associated. Also, Aprotinin was independently predictive of 5-year mortality among patients with diverse risk profiles, as well as those who survived index hospitalization. Neither aminocaproic acid nor tranexamic acid was so associated (Mangano, 2007). The conclusions of the study are clear:

These findings indicate that in addition to the previously reported acute renal and vascular safety concerns, aprotinin use is associated with an increased risk of long-term mortality following CABG surgery. Use of aprotinin among patients undergoing CABG surgery does not appear prudent because safer and less expensive alternatives (i.e., aminocaproic acid and tranexamic acid) are available (Mangano, 2007).

Why then did Bayer not reveal its own data earlier, which also found major problems with the drug? Why did it have to promote the drug in spite of such data in its possession? The reason is the new drug had to be marketed and marketed more than 100 times costlier, oblivious of data with the company that it had serious side effects. Because the profits had to be made and that motive rode roughshod over all other considerations. (Trasylol is much costlier than both the drugs used for the same condition hitherto. Aminocaproic acid and tranexamic acid cost $11 and $44 per dose, respectively, compared to $1,300 per dose of Trasylol.)

Probably, some smart marketing man or group prevailed over safety and scientific considerations.

That's why the image of pharma stinks. That's when it raises an unbearable stench.

This is just one recent example of a phenomenon, fairly rampant today and much in need of repair, if industry has to salvage any of its lost prestige and credibility.

## Undisclosed Payments to Researchers and Spokespersons

Another area of concern, which does no good either to pharma image or to that of researchers associated with it, is undisclosed financial relationships. A recent example which came to light is the acknowledgement by a federal research scientist working for the National Institute Of Mental Health that he had not disclosed to his employers about 300,000 $ he received from Pfizer as consulting fees and expense payments. It all came to light following whistle blowing by a fellow researcher over 25 diverted samples of CSF. It is reported in the 16^th^ Dec 2006 issue of the *BMJ* (Lenzer, 2006).

While this puts the researcher's activities under the scanner, the acts of the pharma company, which paid such huge sums for the diversion of samples (and did not insist on disclosure), cannot be condoned. Such revelations only add to the murky image of a pharma which makes such large payments and thereby encourages concealment in its associated researchers.

One can recount further tales of such misdemeanors. But they would only add darkness to the already black. What needs to be put into place urgently is a strict enforcement of conflict of interest (not just *declaration*, but *enforcement*) and greater courage by potential whistleblowers. The need to promote biomedical advance and protect research integrity must prevail upon concerns related to protection of fellow researchers and practitioners. For Industry, it is necessary to prevail upon connected researchers to disclose financial conflicts of interest and not engage them in activities that tempt them to conceal such conflicts. Till such time, concerned medical journalists and ethically conscious researchers and journal editors, must keep their critical antennae up and maintain a constant vigil.

## Salvaging AMC Credibility

Salvaging of credibility is not only the concern of Industry. It is also the concern of Academic Medical Centers (AMCs), which, we know, are involved with industry to a large extent and benefiting enormously from such involvement. A recent policy proposal to minimize conflict of interest of AMCs (Brennen *et al*, 2006) notes that while Industry promotes patient welfare by their commitment to research and product development and makes investments in discovering, developing and distributing new pharmaceutical agents, and while they also support continuing medical education, their ultimate fiduciary responsibility is to their shareholders who expect reasonable returns on profits. Also, manufacturers are acutely aware of the conflict between patient vulnerability and profit motives (ibid), and whenever there is such a conflict, industry is often inclined to tilt towards the latter.

Also, recent US congressional investigations, federal prosecutions and class action lawsuits have brought to light documents demonstrating how company practices frequently cross the line between patient welfare and profit-seeking behaviour (Brennen *et al.*, 2006). A recent example of that is the case of Aprotinin we detailed earlier. It is only one more affirmation of how company practices frequently cross the line between patient welfare and profit seeking. Not the first, and certainly not the last, of such practices. Concerned physicians, journalists and federal agencies are exposing still other aspects of an unhealthy relationship between manufacturers and the medical profession (ibid). (And these journalists include medical journalists too.)

AMCs in the US and other medical colleges and institutes elsewhere, need to take the lead in eliminating pervasive conflicts of interest. They can do so by more stringent regulation and regulation/modification of some common practices in AMCs and medical colleges/institutes:

More stringent regulation is necessary, including the elimination or modification of common practices related to small gifts, pharmaceutical samples, continuing medical education, funds for physician travel, speakers bureaus, ghostwriting and consulting and research contracts. We propose a policy under which academic medical centers would take the lead in eliminating the conflicts of interest that still characterize the relationship between physicians and the health care industry (Brennen et al., 2006).

While these measures are worth a close look, we must also realise that the constant pampering by sponsors has dulled most drug prescribers′ critical capabilities. Sponsorship is a potent anaesthetic to many ethical concerns. It can blunt the thrust of many a sabre-rattling critic.

## Concluding Remarks

It is obvious, then, that this is a protracted unrelenting struggle, only likely to abate if all parties concerned look to their long-term welfare and accept the win-win proposal for them we outline elsewhere (Singh and Singh, 2007; p11-14). However, let's note that most such considerations are heard and implemented only when they get well and truly cornered and find no way out. Let's hope they either get cornered fast, or listen to reason before they are subjected to such cornering. Lest you feel skeptical, do note that life, and even its most greed-motivated practitioners, has the uncanny ability to sometimes spring the most pleasant surprises.
